# Immune Background Profiles Across B-cell Lymphoproliferative Disorders

**DOI:** 10.7759/cureus.117134

**Published:** 2026-09-29

**Authors:** Thushara Balasuriya, Chamarika Moonesinghe, Neluka Fernando, Chinthika Gunasekara, Surangi Jayakody, Chitranga C Kariyawasan

**Affiliations:** 1 Hematology, Sri Jayewardenepura General Hospital, Colombo, LKA; 2 Hematology, University of Sri Jayewardenepura, Nugegoda, LKA; 3 Microbiology, University of Sri Jayewardenepura, Nugegoda, LKA; 4 Warwick Medical School, University of Warwick, Coventry, GBR

**Keywords:** b-cell lymphoproliferative disorders, b cells, flow cytometry, immune profile, lymphocyte subsets, natural killer cells, t cells

## Abstract

Introduction

Flow cytometry is essential for the diagnosis and classification of B-cell chronic lymphoproliferative disorders (B-CLPDs), but routine assessment usually focuses on the clonal B-cell population. Background T-cell and natural killer (NK)-cell populations may also vary between subtypes and provide supportive immunological information. Peripheral blood and bone marrow samples should be analyzed separately because they represent biologically distinct compartments. This study compares the percentages and absolute counts of B cells, T cells, CD4-positive T cells, CD8-positive T cells, and NK cells across B-CLPD subtypes, with analyses stratified by sample type where appropriate.

Methods

The study included 210 newly diagnosed B-CLPD cases evaluated at the Department of Hematology, Sri Jayewardenepura General Hospital, Colombo, Sri Lanka, from July 2023 to December 2025. Diagnoses were based on morphology and multiparameter flow cytometric immunophenotyping. CD19+ B cells, CD3+ T cells, CD3+CD4+ T cells, CD3+CD8+ T cells, CD56+ NK cells, and the CD4/CD8 ratio were analyzed. Results were summarized using medians and interquartile ranges, and subtype differences were assessed using the Kruskal-Wallis test.

Results

Of the 210 cases, 181 were peripheral blood samples and 29 were bone marrow samples. In peripheral blood, B-cell, T-cell, and NK-cell measures differed significantly across B-CLPD subtypes. Significant differences were also observed in CD4+ T-cell count, CD4+ T-cell percentage, CD8+ T-cell count, and the CD4/CD8 ratio, whereas CD8+ T-cell percentage did not differ significantly. In the bone marrow, no significant subtype-related differences were observed in the CD4/CD8-related measures examined.

Conclusion

B-CLPD subtypes show distinct immune profiles, particularly in peripheral blood. Routine lymphocyte subset data may provide useful supportive information during flow-cytometric assessment, although they should not replace established lineage- and subtype-specific diagnostic markers.

## Introduction

B-cell chronic lymphoproliferative disorders (B-CLPDs) comprise a biologically heterogeneous group of mature B-cell neoplasms, including chronic lymphocytic leukemia (CLL), mantle cell lymphoma (MCL), follicular lymphoma (FL), splenic marginal zone lymphoma (SMZL), lymphoplasmacytic lymphoma (LPL), hairy cell leukemia (HCL), and related entities. Current disease classifications emphasize an integrated diagnostic approach based on morphology, immunophenotype, clinical context, and ancillary laboratory findings because several mature B-cell neoplasms share overlapping features [1,2].

Multiparameter flow cytometry has an established role in the rapid evaluation of mature B-cell neoplasms, particularly in peripheral blood and bone marrow samples. It confirms clonality, identifies lineage-defining patterns, and helps distinguish closely related disorders through combinations of backbone and discriminatory markers [3,4].

Most diagnostic flow cytometric assessments in B-CLPDs focus primarily on the neoplastic B-cell population. However, the background immune compartment is also clinically relevant. The B-cell compartment reflects the burden and distribution of the clonal population and remains central to lineage confirmation, clonality assessment, and estimation of circulating disease burden [3,4]. The total T-cell compartment provides broader information on host immune status and tumor-immune interactions, while natural killer (NK) cells are key mediators of innate antitumor surveillance and may be quantitatively or functionally altered in mature B-cell neoplasms, particularly in CLL [5-9]. Therefore, variation in B-cell, T-cell, and NK-cell proportions across B-CLPD subtypes may have biological and supportive interpretive value during routine flow cytometric assessment.

CD4-positive and CD8-positive T-cell subsets may provide a more detailed view of the background immune compartment. CD4-positive T cells contribute to helper and regulatory immune functions, whereas CD8-positive T cells represent the main cytotoxic effector subset involved in antitumor immune responses. Changes in their relative proportions and in the CD4/CD8 ratio may therefore reflect immune imbalance, chronic antigenic stimulation, or T-cell dysfunction [8-13]. This may be especially relevant in CLL, where expansion of dysfunctional or exhausted CD8-positive T cells and inversion of the CD4/CD8 ratio have been linked to disease biology and prognosis [10-13]. Interpretation should also consider sample type because peripheral blood and bone marrow are biologically distinct compartments. Published data indicate that bone marrow CD4/CD8 ratios can be substantially lower than peripheral blood ratios and that marrow lymphocyte composition may be influenced by compartment-specific microenvironmental factors and hemodilution of aspirates [14,15].

In resource-limited settings, obtaining additional information from routine diagnostic flow cytometry is particularly valuable because it does not require further specialized testing. Accordingly, this descriptive, hypothesis-generating study had two main objectives: (i) to compare the percentages and absolute counts of B cells, T cells, and NK cells across B-CLPD subtypes; and (ii) to compare CD4-positive and CD8-positive T-cell counts, percentages, and the CD4/CD8 ratio across B-CLPD subtypes, with peripheral blood and bone marrow analyzed separately. The study was intended to describe subtype-associated immune background patterns rather than to establish predictive or causal relationships.

## Materials and methods

Study design and setting

This descriptive, hypothesis-generating study was conducted at the Department of Hematology, Sri Jayewardenepura General Hospital, Colombo, Sri Lanka, a tertiary referral center for hematological malignancies. The study period extended from July 2023 to December 2025. The parent diagnostic cohort included 210 consecutive newly diagnosed B-CLPD cases.

Study population

Only cases with a confirmed diagnosis based on integrated morphological assessment and multiparameter flow cytometric immunophenotyping were considered eligible. Patients who were receiving chemotherapy or immunotherapy at the time of sampling, cases with inadequate sample quality, and cases with incomplete clinical or laboratory data were excluded.

Clinical assessment and specimen handling

At diagnosis, patients underwent a structured clinical assessment. Clinical findings, including pallor, persistent lymphocytosis, splenomegaly, hepatomegaly, lymphadenopathy, and fever, were documented from clinical records. Peripheral blood collected in EDTA-anticoagulated tubes was used for diagnostic evaluation in most cases. Bone marrow aspirate samples were obtained when peripheral blood findings did not permit a definitive diagnosis or when bone marrow assessment was required for confirmation or staging. All samples were processed immediately according to standard laboratory operating procedures.

Full blood count and morphology

Full blood counts in the study cohort were performed using the Mindray BC-6800 Plus fully automated hematology analyzer (Shenzhen Mindray Bio-Medical Electronics Co., Ltd., Shenzhen, China). Cytomorphological evaluation was performed on Leishman-stained peripheral blood and/or bone marrow aspirate smears by experienced hematology personnel. Morphological assessment included the nuclear-to-cytoplasmic ratio, nuclear contour, nucleolar prominence, and cell size. These findings were interpreted together with the immunophenotypic profile to support subtype classification.

Flow-cytometric immunophenotyping

Flow cytometry was performed using a BD FACSCanto II flow cytometer (BD Biosciences, San Jose, CA, USA), and data acquisition and analysis were carried out using BD FACSDiva software (BD Biosciences, San Jose, CA, USA). Multiparameter staining was performed on peripheral blood or bone marrow aspirate samples using standardized protocols aligned with EuroFlow recommendations and manufacturer-validated procedures. The standard diagnostic antibody panel included CD45 (V500-C), CD19 (PE-Cy7), CD20 (V450), CD5 (PerCP-Cy5.5), CD10 (PE), surface immunoglobulin kappa (PE) and lambda (FITC), CD23 (FITC), CD79b (PerCP-Cy5.5), CD200 (APC), CD38 (APC-H7), CD43 (APC-H7), and FMC7 (FITC), with additional markers applied when clinically indicated. Final subtype classification was based on integrated morphological and immunophenotypic interpretation in accordance with accepted diagnostic criteria for mature B-cell neoplasms [1-4].

Lymphocyte subsets

Lymphocyte subset percentage analysis was performed according to the manufacturer's protocol. First, the absolute white blood cell (WBC) count of each sample was measured using the Mindray BC-6800 Plus hematology analyzer. Samples were then analyzed by flow cytometry using BD OneFlow LST tubes (BD Biosciences, San Jose, CA, USA) following the wash-wash-wash-sample-lyse method. Lymphocyte subset percentages were obtained from flow cytometric analysis, and the corresponding absolute lymphocyte subset counts were calculated using the absolute WBC count and the relevant subset percentages.

All data were generated on the same BD FACSCanto II using the same manufacturer-recommended protocol, with CS&T beads, BD OneFlow Setup Beads, BD OneFlow FC Beads, and same-lot, same-conjugate reagents used throughout the study period to minimize analytical variation.

Study variables

The variables analyzed for the present objective were CD19-gated B-cell count, CD19-gated B-cell percentage, CD3-gated T-cell count, CD3-gated T-cell percentage, CD3+CD4+ T-cell count, CD3+CD4+ T-cell percentage, CD3+CD8+ T-cell count, CD3+CD8+ T-cell percentage, CD4/CD8 ratio, CD56-gated NK-cell count, and CD56-gated NK-cell percentage.

The total WBC count was obtained from the Mindray BC-6800 Plus hematology analyzer. The lymphocyte percentage was determined by flow cytometry from the CD45-gated lymphocyte population, and the absolute lymphocyte count was calculated as follows: absolute lymphocyte count = total WBC count × lymphocyte percentage/100. Absolute counts of B cells, T cells, CD4+ T cells, CD8+ T cells, and NK cells were then calculated from the absolute lymphocyte count using the corresponding flow cytometric subset percentages.

Statistical analysis

Statistical analysis of the study cohort was performed using IBM SPSS Statistics for Windows, Version 20 (Released 2011; IBM Corp., Armonk, NY, USA), and R software (R Foundation for Statistical Computing, Vienna, Austria). Continuous variables were assessed for their distribution. Because lymphocyte subset data were skewed and subgroup sizes were unequal, results were summarized using medians and interquartile ranges. The B-cell, T-cell, and NK-cell analyses were based on peripheral blood-derived subset data. CD4-positive and CD8-positive T-cell variables were analyzed separately for peripheral blood and bone marrow samples to avoid pooling biologically distinct specimen types. Differences across disease categories were assessed using the Kruskal-Wallis test. Because the hairy cell leukemia variant (HCL-v) subgroup contained only three cases overall, it was retained for descriptive reporting but excluded from the main peripheral blood omnibus inferential comparison. In the bone marrow subset, inferential testing was restricted to groups with at least three cases. A p-value of <0.05 was considered statistically significant.

Ethical considerations

Ethical approval was obtained from the Ethics Review Committee of the University of Sri Jayewardenepura (ERC 08/23). All investigations were performed as part of routine diagnostic evaluation, and patient confidentiality was strictly maintained.

## Results

Cohort overview

A total of 210 B-CLPD cases were included in the study cohort. CLL was the most common subtype (94/210, 44.8%), followed by MCL (34/210, 16.2%) and CD5-negative/CD10-negative/hairy-marker-negative B-CLPD (CD5-/CD10-/hairy-marker- B-CLPD) (31/210, 14.8%). Smaller subgroups included FL, B-PLL, LPL, HCL, SMZL, DLBCL, atypical CLL, and HCL-v.

The baseline distribution of B-CLPD subtypes, including age and sex profiles, is summarized in Table 1.

**Table 1:** Baseline subtype distribution with age and sex profile CD5-/CD10-/hairy-B-CLPD, CD5-negative, CD10-negative, hairy-cell-marker-negative B-cell chronic lymphoproliferative disorder; CLL, chronic lymphocytic leukemia; MCL, mantle cell lymphoma; LPL, lymphoplasmacytic lymphoma; B-PLL, B-cell prolymphocytic leukemia; HCL, hairy cell leukemia; HCL-v, hairy cell leukemia variant; SMZL, splenic marginal zone lymphoma; FL, follicular lymphoma; DLBCL, diffuse large B-cell lymphoma; IQR, interquartile range

Subtype	n	Male, n (%)	Age, median (IQR)
CD5-/CD10-/hairy-B-CLPD	31	15 (48.4)	69.0 (55.5-76.5)
CLL	94	57 (60.6)	66.0 (59.0-71.8)
MCL	34	26 (76.5)	73.0 (67.2-76.8)
LPL	7	5 (71.4)	66.0 (62.0-68.5)
B-PLL	8	4 (50.0)	79.0 (72.0-80.0)
HCL	7	6 (85.7)	60.0 (53.0-65.5)
HCL-v	3	3 (100.0)	55.0 (54.0-66.5)
SMZL	6	4 (66.7)	82.0 (76.8-83.5)
FL	9	6 (66.7)	54.0 (37.0-61.0)
DLBCL	5	4 (80.0)	68.0 (68.0-74.0)
Atypical CLL	6	5 (83.3)	70.0 (69.2-74.5)

The overall distribution of B-CLPD subtypes in the study cohort is illustrated in Figure 1.

**Figure 1:**
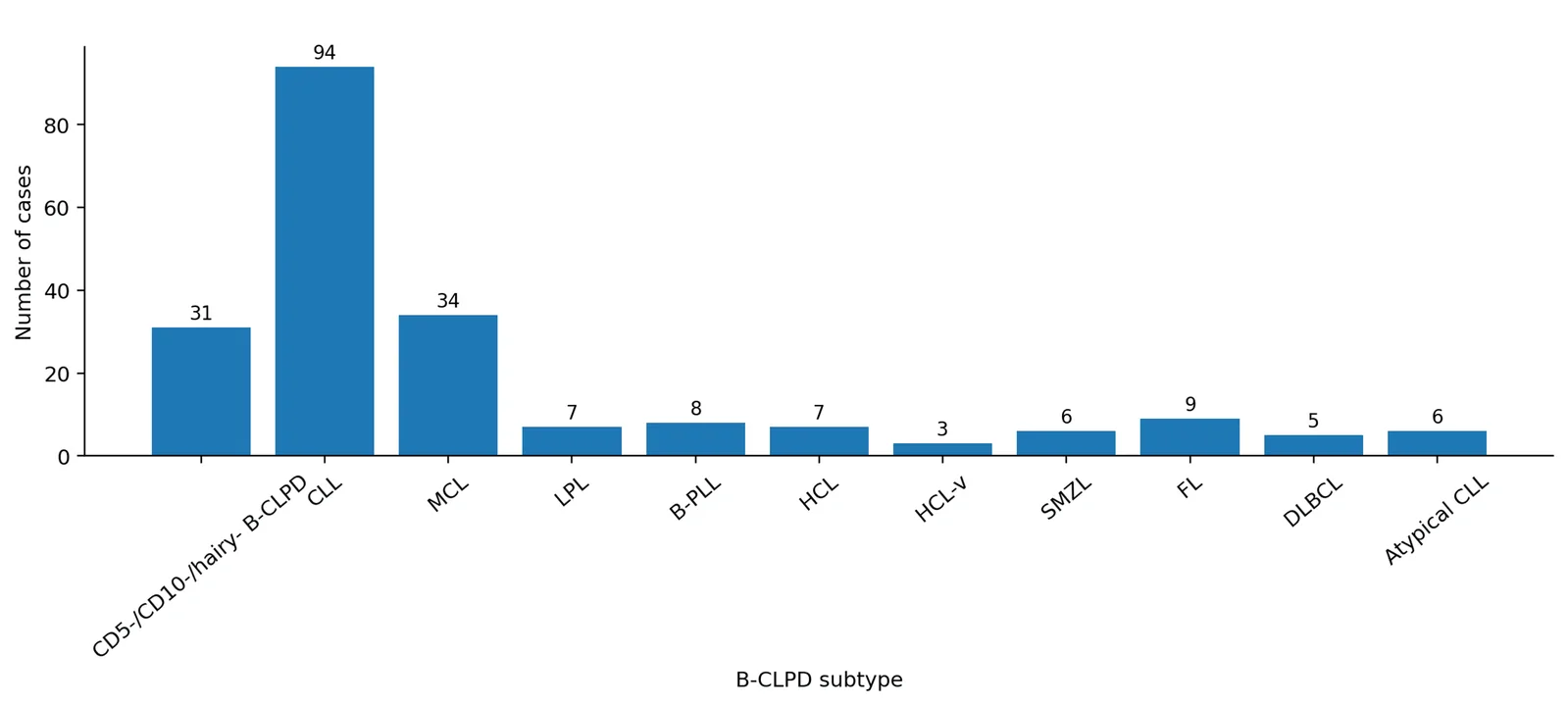
Distribution of B-CLPD subtypes in the study cohort CD5-/CD10-/hairy-B-CLPD, CD5-negative, CD10-negative, hairy-cell-marker-negative B-cell chronic lymphoproliferative disorder; CLL, chronic lymphocytic leukemia; MCL, mantle cell lymphoma; LPL, lymphoplasmacytic lymphoma; B-PLL, B-cell prolymphocytic leukemia; HCL, hairy cell leukemia; HCL-v, hairy cell leukemia variant; SMZL, splenic marginal zone lymphoma; FL, follicular lymphoma; DLBCL, diffuse large B-cell lymphoma

The overall Kruskal-Wallis comparisons of peripheral blood B-cell, T-cell, and NK-cell measures across the major B-CLPD subtypes are presented in Table 2.

**Table 2:** Kruskal-Wallis analysis of lymphocyte subset measures across major B-CLPD subtypes B-CLPD, B-cell chronic lymphoproliferative disorder; NK, natural killer, H, Kruskal-Wallis test statistic

Variable	Kruskal-Wallis H	p-value
B-cell count	44.12	<0.001
B-cell percentage	40.887	<0.001
T-cell count	48.49	<0.001
T-cell percentage	27.962	<0.001
NK-cell count	37.644	<0.001
NK-cell percentage	29.651	<0.001

The median absolute B-cell, T-cell, and NK-cell counts in peripheral blood for each B-CLPD subtype are summarized in Table 3.

**Table 3:** Absolute lymphocyte subset counts by B-CLPD subtype, expressed as median (IQR) CD5-/CD10-/hairy-B-CLPD, CD5-negative, CD10-negative, hairy-cell-marker-negative B-cell chronic lymphoproliferative disorder; CLL, chronic lymphocytic leukemia; MCL, mantle cell lymphoma; LPL, lymphoplasmacytic lymphoma; B-PLL, B-cell prolymphocytic leukemia; HCL, hairy cell leukemia; HCL-v, hairy cell leukemia variant; SMZL, splenic marginal zone lymphoma; FL, follicular lymphoma; DLBCL, diffuse large B-cell lymphoma; NK, natural killer; IQR, interquartile range

Subtype	n	B-cell count	T-cell count	NK-cell count
CD5-/CD10-/hairy-B-CLPD	23	3,072.1 (1,514.5-11,753.4)	1,611.0 (1,135.7-2,938.2)	721.4 (471.0-1,259.2)
CLL	90	8,676.7 (4,909.2-20,300.2)	3,702.8 (2,138.0-5,515.2)	2,034.1 (1,130.8-4,076.8)
MCL	31	7,302.0 (3,762.2-20,487.7)	1,706.0 (1,511.0-3,777.6)	702.0 (586.5-1,481.7)
LPL	4	811.8 (533.8-1,137.4)	1,823.2 (1,360.5-2,435.9)	277.2 (122.9-681.3)
B-PLL	6	80,804.0 (80,804.0-80,804.0)	5,479.0 (5,479.0-5,479.0)	1,739.0 (1,739.0-1,739.0)
HCL	7	1,515.5 (560.1-5,294.1)	920.0 (631.0-1,314.8)	373.0 (334.0-931.6)
HCL-v	2	57,208.7 (49,427.3-64,990.0)	4,774.2 (4,380.3-5,168.1)	1,456.9 (1,158.3-1,755.5)
SMZL	5	12,097.0 (5,442.7-12,832.2)	2,340.4 (1,358.0-3,673.0)	1,249.0 (590.0-1,884.0)
FL	4	60,542.1 (42,120.2-75,341.5)	3,148.7 (2,493.1-3,476.8)	1,137.1 (737.0-2,696.8)
DLBCL	3	11,665.0 (8,021.0-31,106.5)	2,780.4 (1,779.2-3,074.2)	2,942.8 (1,911.4-11,814.5)
Atypical CLL	6	9,384.0 (6,776.5-12,907.3)	1,780.8 (1,490.8-3,032.2)	1,054.3 (746.1-1,867.2)

The corresponding median percentages of peripheral blood B cells, T cells, and NK cells across B-CLPD subtypes are shown in Table 4.

**Table 4:** Lymphocyte subset percentages by B-CLPD subtype, expressed as median (IQR) CD5-/CD10-/hairy-B-CLPD, CD5-negative, CD10-negative, hairy-cell-marker-negative B-cell chronic lymphoproliferative disorder; CLL, chronic lymphocytic leukemia; MCL, mantle cell lymphoma; LPL, lymphoplasmacytic lymphoma; B-PLL, B-cell prolymphocytic leukemia; HCL, hairy cell leukemia; HCL-v, hairy cell leukemia variant; SMZL, splenic marginal zone lymphoma; FL, follicular lymphoma; DLBCL, diffuse large B-cell lymphoma; NK, natural killer; IQR, interquartile range

Subtype	n	B-cell (%)	T-cell (%)	NK-cell (%)
CD5-/CD10-/hairy-B-CLPD	23	55.9 (32.3-81.1)	33.0 (12.9-47.2)	13.6 (8.0-18.0)
CLL	90	69.5 (49.7-76.2)	22.4 (14.4-33.9)	14.6 (9.5-21.4)
MCL	31	76.9 (60.5-76.9)	17.2 (16.5-26.2)	7.4 (5.3-15.8)
LPL	4	20.8 (17.3-25.7)	61.2 (45.2-68.9)	5.5 (3.9-16.1)
B-PLL	6	92.9 (92.9-92.9)	6.3 (6.3-6.3)	2.0 (2.0-2.0)
HCL	7	34.6 (28.1-67.5)	38.6 (21.6-39.1)	24.3 (14.8-32.6)
HCL-v	2	88.1 (85.6-90.6)	8.1 (6.6-9.6)	2.6 (1.9-3.3)
SMZL	5	78.0 (71.7-80.6)	16.5 (14.7-24.2)	9.2 (5.9-12.5)
FL	4	90.4 (87.6-93.3)	4.8 (4.2-6.2)	4.9 (1.1-11.6)
DLBCL	3	75.5 (70.1-83.2)	11.5 (8.2-16.6)	37.2 (21.5-40.4)
Atypical CLL	6	74.1 (67.2-77.8)	18.6 (15.0-21.7)	12.3 (7.8-19.1)

CD4-positive and CD8-positive T-cell subset analysis stratified by sample type

Of the 210 cases, 181 were analyzed using peripheral blood samples and 29 using bone marrow samples. Because these specimen types differ biologically, CD4-positive and CD8-positive T-cell subset analyses were performed separately.

Peripheral blood analysis

In the peripheral blood subset, statistically significant inter-subtype differences were observed in CD4-positive T-cell count, CD4-positive T-cell percentage, CD8-positive T-cell count, and the CD4/CD8 ratio. CD8-positive T-cell percentage did not differ significantly across subtypes. Descriptively, B-PLL showed the highest median absolute CD4-positive and CD8-positive T-cell counts, whereas HCL showed the lowest median absolute counts. The highest median CD4-positive T-cell percentage and CD4/CD8 ratio were observed in the CD5-/CD10-/hairy- B-CLPD group, whereas the highest median CD8-positive T-cell percentage was observed in LPL.

The Kruskal-Wallis results for peripheral blood CD4-positive and CD8-positive T-cell subset measures are presented in Table 5.

**Table 5:** Kruskal-Wallis analysis of peripheral blood CD4-positive and CD8-positive T-cell subset measures across major B-CLPD subtypes CD, cluster of differentiation; B-CLPD, B-cell chronic lymphoproliferative disorder; H, Kruskal-Wallis test statistic

Variable	Kruskal-Wallis H	p-value
CD4-positive T-cell count	35.219	<0.001
CD4-positive T-cell percentage	23.009	0.006
CD8-positive T-cell count	55.139	<0.001
CD8-positive T-cell percentage	13.865	0.127
CD4/CD8 ratio	19.522	0.021

Subtype-specific peripheral blood CD4-positive and CD8-positive T-cell counts, percentages, and CD4/CD8 ratios are summarized in Table 6.

**Table 6:** Peripheral blood CD4-positive and CD8-positive T-cell subset measures by B-CLPD subtype, expressed as median (IQR) CD5-/CD10-/hairy-B-CLPD, CD5-negative, CD10-negative, hairy-cell-marker-negative B-cell chronic lymphoproliferative disorder; CLL, chronic lymphocytic leukemia; MCL, mantle cell lymphoma; LPL, lymphoplasmacytic lymphoma; B-PLL, B-cell prolymphocytic leukemia; HCL, hairy cell leukemia; HCL-v, hairy cell leukemia variant; SMZL, splenic marginal zone lymphoma; FL, follicular lymphoma; DLBCL, diffuse large B-cell lymphoma; CD, cluster of differentiation; IQR, interquartile range

Subtype	n	CD4+ T-cell count	CD4+ T-cell (%)	CD8+ T-cell count	CD8+ T-cell (%)	CD4/CD8 ratio
CD5-/CD10-/hairy-B-CLPD	23	990.3 (439.5-1,864.0)	64.7 (58.0-74.5)	511.0 (381.9-680.5)	32.4 (22.6-38.0)	2.0 (1.6-3.3)
CLL	90	1,925.6 (956.7-3,312.9)	56.0 (48.8-61.7)	1,300.8 (822.5-2,320.2)	40.0 (34.0-44.4)	1.4 (1.1-1.8)
MCL	31	1,209.4 (916.3-2,434.7)	56.1 (51.5-64.8)	684.3 (477.9-1,352.6)	41.9 (32.4-45.2)	1.3 (1.1-2.0)
LPL	4	865.1 (680.1-1,100.2)	47.6 (45.2-50.3)	827.1 (611.0-1,065.1)	43.5 (38.7-45.1)	1.1 (1.1-1.3)
B-PLL	6	3,293.0 (3,293.0-3,293.0)	60.1 (40.1-68.1)	2,271.5 (2,093.0-3,200.0)	38.2 (38.2-38.2)	1.6 (1.6-1.6)
HCL	7	424.1 (281.8-795.0)	46.1 (41.6-53.5)	355.1 (296.8-489.5)	43.0 (40.8-49.3)	1.2 (0.8-1.2)
HCL-v	2	2,659.1 (2,186.7-3,131.6)	53.9 (48.5-59.3)	1,399.3 (1,198.0-1,600.7)	43.1 (37.8-48.4)	1.9 (1.8-1.9)
SMZL	5	1,228.7 (636.7-1,551.0)	52.5 (38.0-55.3)	911.0 (897.0-1,013.4)	43.3 (40.0-58.6)	1.2 (0.6-1.4)
FL	4	1,573.9 (999.2-2,077.3)	52.2 (43.3-60.1)	1,025.3 (639.1-1,442.7)	41.8 (29.0-53.6)	1.4 (0.8-2.1)
DLBCL	3	1,044.0 (697.8-1,314.4)	45.2 (38.1-51.1)	1,156.7 (734.4-1,677.8)	41.6 (40.9-53.5)	1.1 (0.8-1.2)
Atypical CLL	6	1,018.0 (934.5-1,603.9)	58.2 (48.5-70.1)	866.5 (551.8-997.5)	35.4 (27.8-47.8)	1.7 (1.0-2.6)

Bone marrow analysis

In the bone marrow subset, no statistically significant inter-subtype differences were observed in CD4-positive T-cell count, CD4-positive T-cell percentage, CD8-positive T-cell count, CD8-positive T-cell percentage, or the CD4/CD8 ratio. These findings should be interpreted cautiously because the bone marrow analysis was limited by small sample sizes, with several subgroups containing only one or two cases.

The Kruskal-Wallis results for bone marrow CD4-positive and CD8-positive T-cell subset measures are presented in Table 7.

**Table 7:** Kruskal-Wallis analysis of bone marrow CD4-positive and CD8-positive T-cell subset measures across B-CLPD subtypes CD, cluster of differentiation; B-CLPD, B-cell chronic lymphoproliferative disorder; H, Kruskal-Wallis test statistic

Variable	Kruskal-Wallis H	p-value
CD4-positive T-cell count	8.032	0.09
CD4-positive T-cell percentage	2.505	0.644
CD8-positive T-cell count	4.496	0.343
CD8-positive T-cell percentage	2.54	0.638
CD4/CD8 ratio	3.457	0.484

Subtype-specific bone marrow CD4-positive and CD8-positive T-cell counts, percentages, and CD4/CD8 ratios are summarized in Table 8.

**Table 8:** Bone marrow CD4-positive and CD8-positive T-cell subset measures by B-CLPD subtype, expressed as median (IQR) CD5-/CD10-/hairy- B-CLPD, CD5-negative, CD10-negative, hairy-cell-marker-negative B-cell chronic lymphoproliferative disorder; CLL, chronic lymphocytic leukemia; MCL, mantle cell lymphoma; LPL, lymphoplasmacytic lymphoma; B-PLL, B-cell prolymphocytic leukemia; HCL, hairy cell leukemia; HCL-v, hairy cell leukemia variant; SMZL, splenic marginal zone lymphoma; FL, follicular lymphoma; DLBCL, diffuse large B-cell lymphoma; CD, cluster of differentiation; IQR, interquartile range

Subtype	n	CD4+ T-cell count	CD4+ T-cell (%)	CD8+ T-cell count	CD8+ T-cell (%)	CD4/CD8 ratio
CD5-/CD10-/hairy-B-CLPD	8	720.0 (516.0-1,296.9)	56.5 (42.6-59.1)	499.0 (358.5-1,211.8)	40.9 (35.4-48.6)	1.4 (0.9-1.7)
CLL	4	2,419.5 (1,460.0-4,708.2)	59.2 (58.1-61.1)	1,528.0 (945.2-2,670.8)	37.5 (36.1-39.3)	1.6 (1.5-1.7)
MCL	3	2,088.0 (1,429.0-2,100.5)	49.5 (47.9-53.6)	1,374.0 (1,110.2-1,554.0)	41.1 (39.4-46.0)	1.2 (1.1-1.4)
LPL	3	971.0 (796.5-1,224.7)	40.0 (37.3-51.4)	1,606.0 (946.5-1,727.0)	50.0 (39.5-53.6)	0.8 (0.7-1.5)
B-PLL	2	959.4 (892.2-1,026.6)	49.6 (43.6-55.6)	962.1 (792.7-1,131.5)	47.2 (41.1-53.2)	1.2 (0.9-1.5)
HCL-v	1	1,287.0 (1,287.0-1,287.0)	42.9 (42.9-42.9)	1,266.0 (1,266.0-1,266.0)	42.2 (42.2-42.2)	1.0 (1.0-1.0)
SMZL	1	592.3 (592.3-592.3)	48.1 (48.1-48.1)	555.3 (555.3-555.3)	45.1 (45.1-45.1)	1.1 (1.1-1.1)
FL	5	3,470.0 (2,620.8-4,957.0)	66.6 (37.7-74.7)	1,264.5 (1,172.7-1,277.8)	29.8 (14.7-40.8)	2.2 (1.3-5.1)
DLBCL	2	1,697.5 (1,167.3-2,227.8)	35.3 (34.4-36.2)	2,644.7 (1,876.5-3,412.8)	57.2 (56.8-57.7)	0.6 (0.6-0.7)

The median peripheral blood lymphocyte subset percentages across B-CLPD subtypes are displayed in Figure 2.

**Figure 2:**
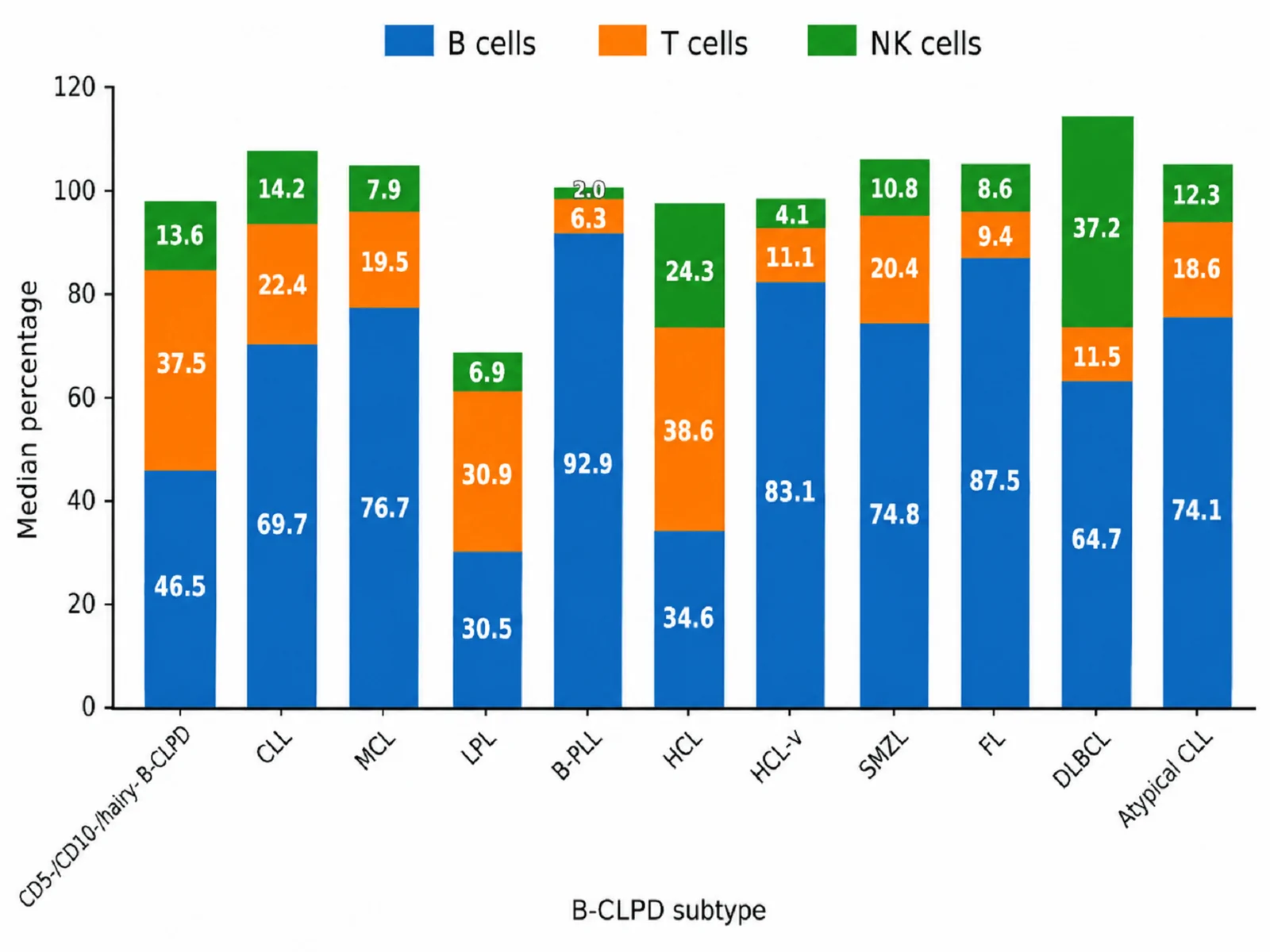
Median peripheral blood lymphocyte subset percentages by subtype CD5-/CD10-/hairy-B-CLPD, CD5-negative, CD10-negative, hairy-cell-marker-negative B-cell chronic lymphoproliferative disorder; CLL, chronic lymphocytic leukemia; MCL, mantle cell lymphoma; LPL, lymphoplasmacytic lymphoma; B-PLL, B-cell prolymphocytic leukemia; HCL, hairy cell leukemia; HCL-v, hairy cell leukemia variant; SMZL, splenic marginal zone lymphoma; FL, follicular lymphoma; DLBCL, diffuse large B-cell lymphoma; NK, natural killer

The overall pattern of median lymphocyte subset measures across B-CLPD subtypes is visualized as a heatmap in Figure 3.

**Figure 3:**
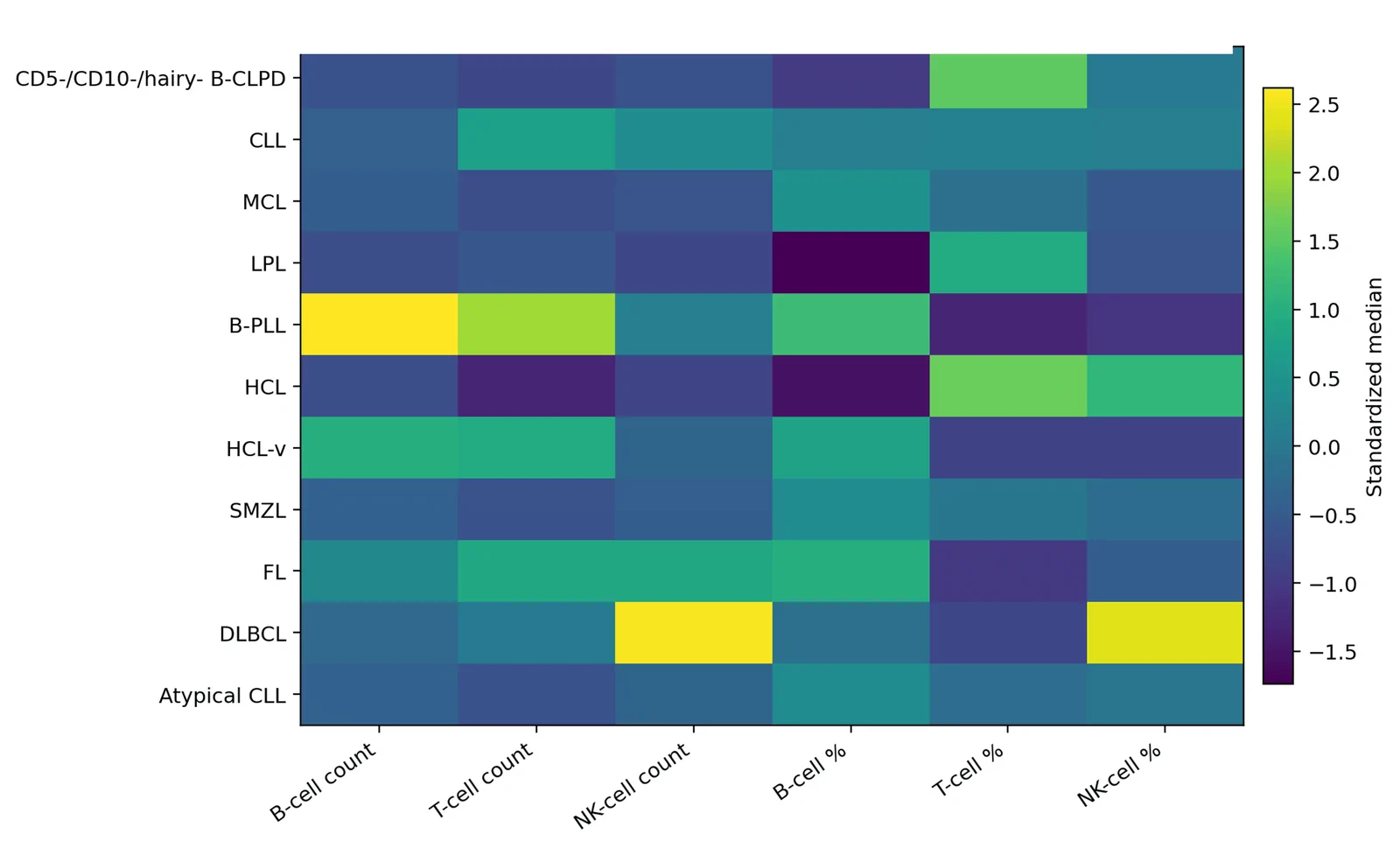
Heatmap of median lymphocyte subset measures across B-CLPD subtypes CD5-/CD10-/hairy-B-CLPD, CD5-negative, CD10-negative, hairy-cell-marker-negative B-cell chronic lymphoproliferative disorder; CLL, chronic lymphocytic leukemia; MCL, mantle cell lymphoma; LPL, lymphoplasmacytic lymphoma; B-PLL, B-cell prolymphocytic leukemia; HCL, hairy cell leukemia; HCL-v, hairy cell leukemia variant; SMZL, splenic marginal zone lymphoma; FL, follicular lymphoma; DLBCL, diffuse large B-cell lymphoma; NK, natural killer

The distribution of absolute B-cell counts across B-CLPD subtypes is shown in Figure 4.

**Figure 4:**
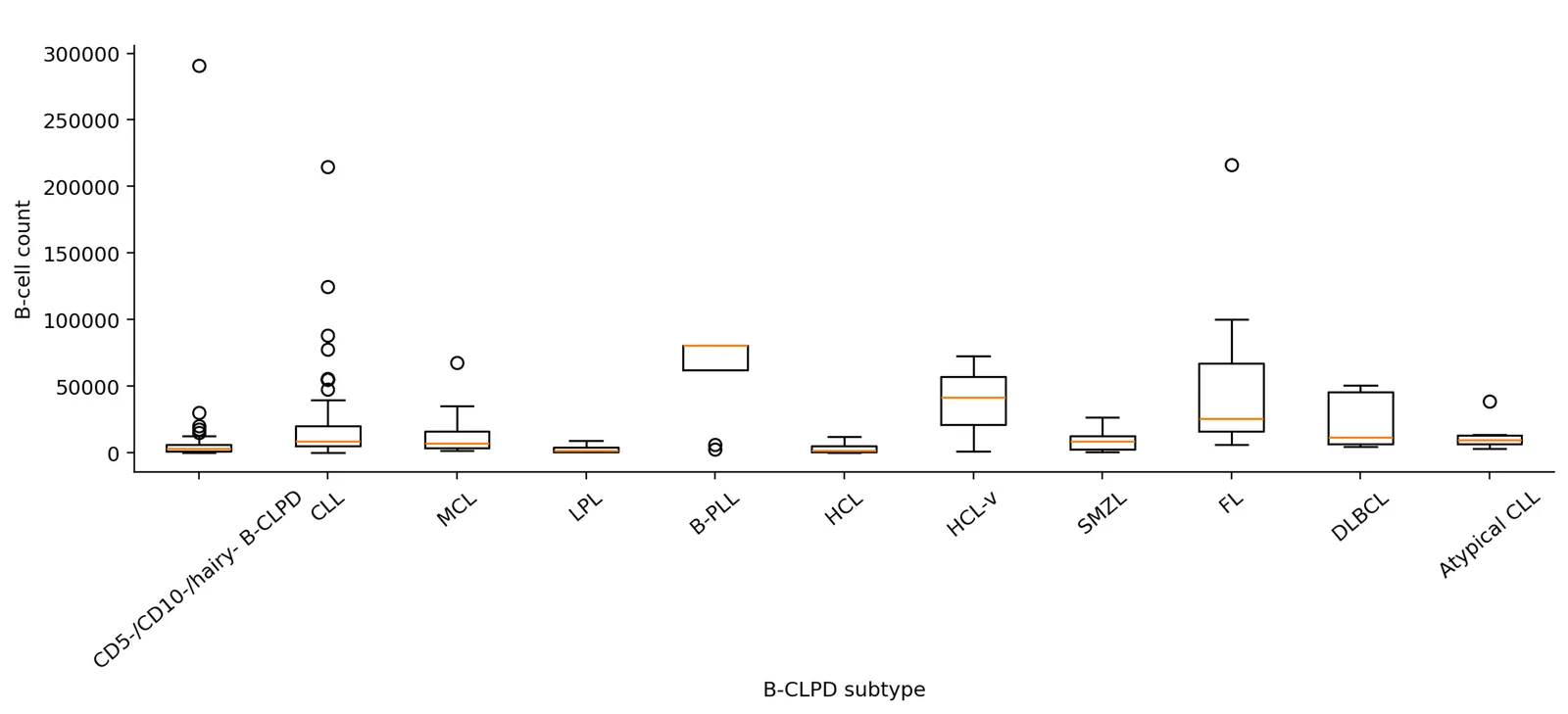
Absolute B-cell counts across B-CLPD subtypes CD5-/CD10-/hairy-B-CLPD, CD5-negative, CD10-negative, hairy-cell-marker-negative B-cell chronic lymphoproliferative disorder; CLL, chronic lymphocytic leukemia; MCL, mantle cell lymphoma; LPL, lymphoplasmacytic lymphoma; B-PLL, B-cell prolymphocytic leukemia; HCL, hairy cell leukemia; HCL-v, hairy cell leukemia variant; SMZL, splenic marginal zone lymphoma; FL, follicular lymphoma; DLBCL, diffuse large B-cell lymphoma

The distribution of absolute T-cell counts across B-CLPD subtypes is shown in Figure 5.

**Figure 5:**
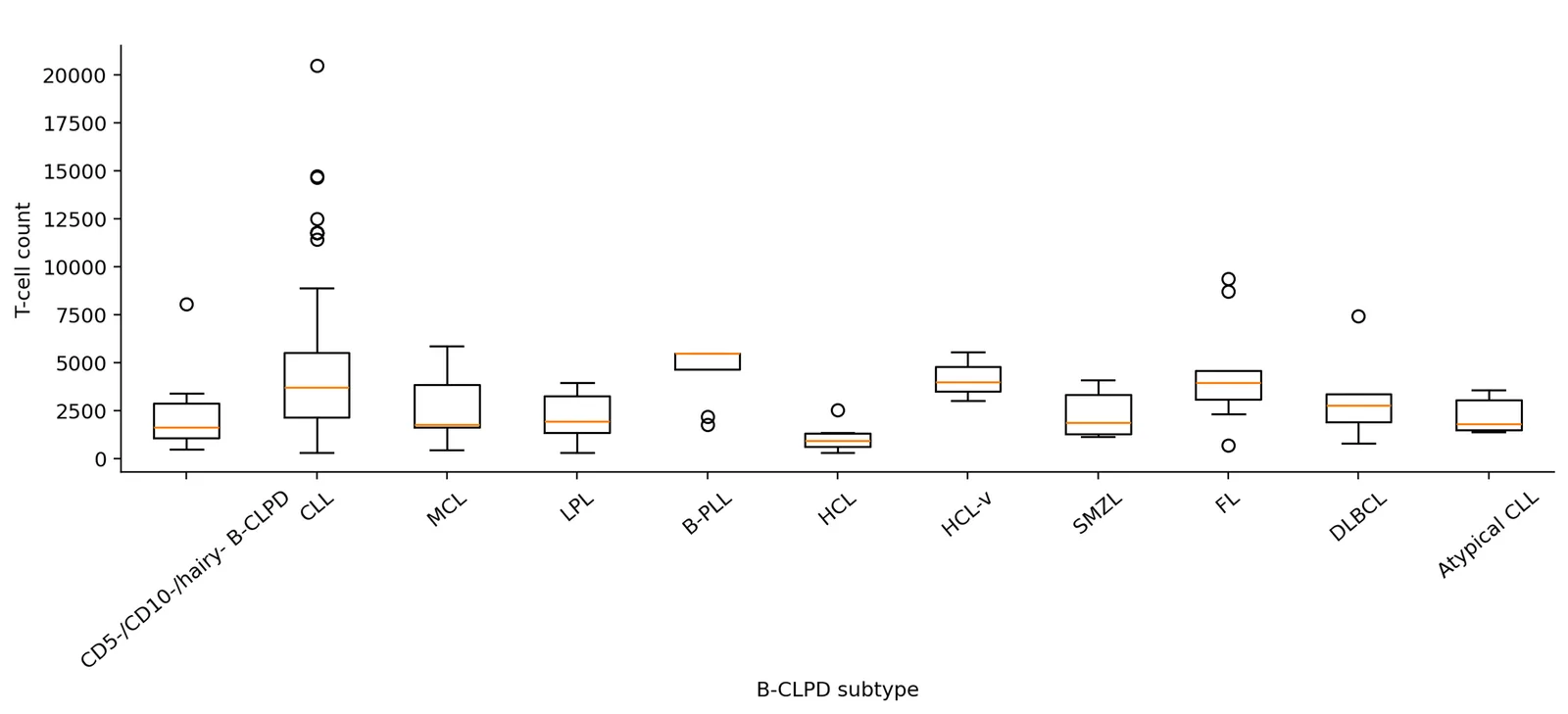
Boxplot of absolute T-cell count across B-CLPD subtypes CD5-/CD10-/hairy-B-CLPD, CD5-negative, CD10-negative, hairy-cell-marker-negative B-cell chronic lymphoproliferative disorder; CLL, chronic lymphocytic leukemia; MCL, mantle cell lymphoma; LPL, lymphoplasmacytic lymphoma; B-PLL, B-cell prolymphocytic leukemia; HCL, hairy cell leukemia; HCL-v, hairy cell leukemia variant; SMZL, splenic marginal zone lymphoma; FL, follicular lymphoma; DLBCL, diffuse large B-cell lymphoma

The distribution of absolute NK-cell counts across B-CLPD subtypes is shown in Figure 6.

**Figure 6:**
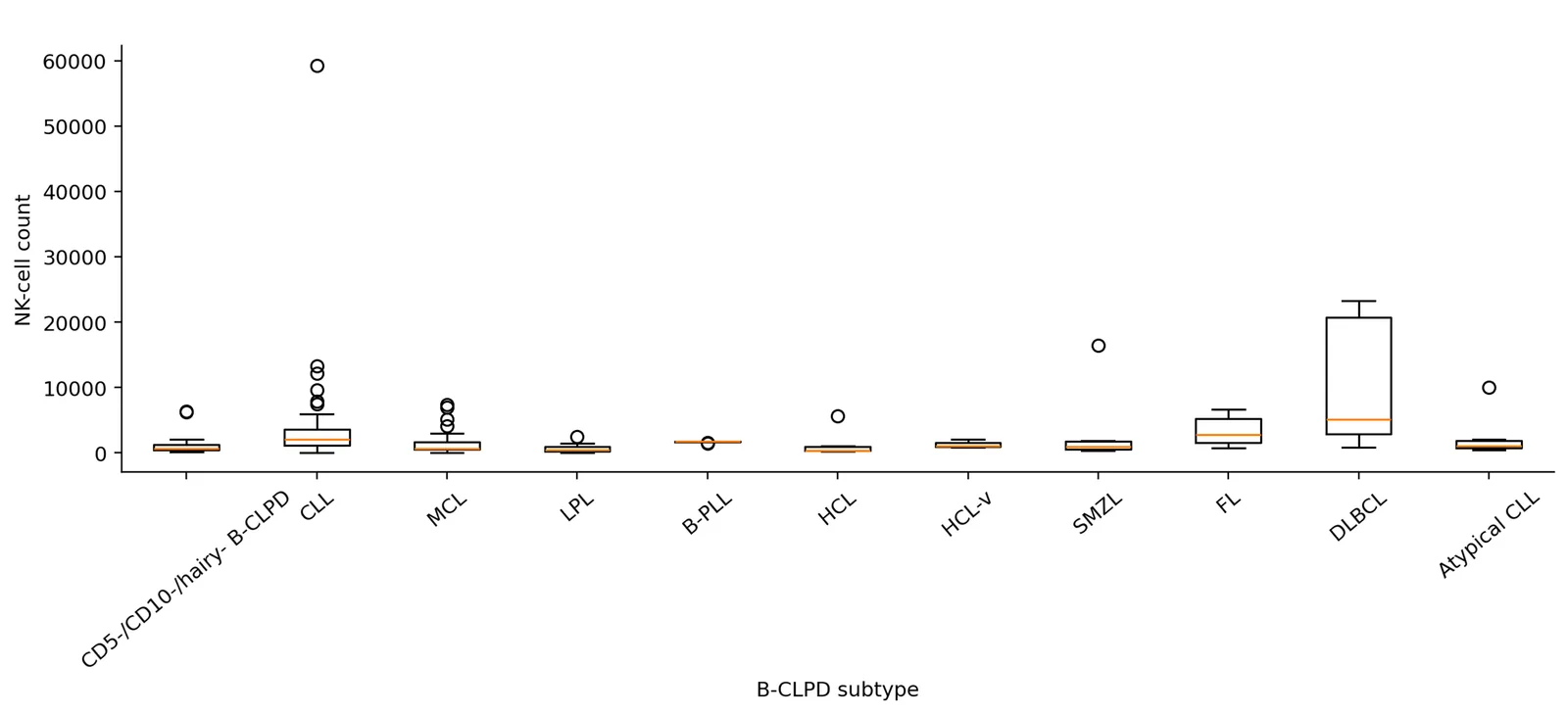
Boxplot of absolute NK-cell count across B-CLPD subtypes CD5-/CD10-/hairy-B-CLPD, CD5-negative, CD10-negative, hairy-cell-marker-negative B-cell chronic lymphoproliferative disorder; CLL, chronic lymphocytic leukemia; MCL, mantle cell lymphoma; LPL, lymphoplasmacytic lymphoma; B-PLL, B-cell prolymphocytic leukemia; HCL, hairy cell leukemia; HCL-v, hairy cell leukemia variant; SMZL, splenic marginal zone lymphoma; FL, follicular lymphoma; DLBCL, diffuse large B-cell lymphoma; NK, natural killer

## Discussion

This study showed that lymphocyte subset distributions vary across B-CLPDs and that these differences are more apparent when peripheral blood samples are analyzed separately. Significant variation was observed not only in the overall B-cell, T-cell, and NK-cell compartments but also in CD4-positive T-cell count, CD4-positive T-cell percentage, CD8-positive T-cell count, and the CD4/CD8 ratio in peripheral blood. These findings suggest that the immune background differs across B-CLPD subtypes and may reflect differences in tumor burden and host immune response [5-13].

The marked B-cell predominance observed in B-PLL and FL is biologically plausible. B-PLL is a leukemic mature B-cell neoplasm associated with a high circulating tumor burden, whereas CLL is characterized by persistent clonal B-cell lymphocytosis in peripheral blood [3,16]. Therefore, the high B-cell proportions observed in these subtypes are consistent with their underlying disease biology. In contrast, variation in T-cell and NK-cell measures suggests that the non-neoplastic immune compartment may also differ across subtypes and may be influenced by the extent of clonal B-cell expansion [3,4,16].

The peripheral blood CD4-positive and CD8-positive T-cell findings are also relevant. In CLL, previous studies have demonstrated quantitative and functional abnormalities in T cells, including chronic activation, exhaustion, impaired immune synapse formation, and expansion of dysfunctional cytotoxic populations [6,8-13]. Inversion of the CD4/CD8 ratio has also been associated with disease biology and prognosis in some studies [10-13]. In this context, the observed differences in CD4-positive T-cell count, CD8-positive T-cell count, and the CD4/CD8 ratio across B-CLPD subtypes suggest that routine lymphocyte subset data may provide supportive information regarding immune disturbances in these disorders [6,8-13].

The relatively higher T-cell and NK-cell proportions observed in HCL were also notable. Classical HCL often presents with cytopenias and splenomegaly rather than marked circulating B-cell predominance, which may partly explain the relatively greater contribution of background lymphocyte subsets in peripheral blood [2,4]. In addition, HCL is strongly dependent on the bone marrow and splenic microenvironment for tumor-cell survival and retention [17-19]. Therefore, the pattern observed in this study may reflect both a lower circulating tumor burden and the contribution of tissue-based microenvironmental support in HCL [17-19].

This study showed that lymphocyte subset distributions vary across B-CLPDs and that these differences are more apparent when peripheral blood samples are analyzed separately. Significant variation was observed not only in the overall B-cell, T-cell, and NK-cell compartments but also in CD4-positive T-cell count, CD4-positive T-cell percentage, CD8-positive T-cell count, and the CD4/CD8 ratio in peripheral blood. These findings suggest that the immune background differs across B-CLPD subtypes and may reflect differences in tumor burden and host immune response [5-13].

The marked B-cell predominance observed in B-PLL and FL is biologically plausible. B-PLL is a leukemic mature B-cell neoplasm associated with a high circulating tumor burden, whereas CLL is characterized by persistent clonal B-cell lymphocytosis in peripheral blood [3,16]. Therefore, the high B-cell proportions observed in these subtypes are consistent with their underlying disease biology. In contrast, variation in T-cell and NK-cell measures suggests that the non-neoplastic immune compartment may also differ across subtypes and may be influenced by the extent of clonal B-cell expansion [3,4,16].

The peripheral blood CD4-positive and CD8-positive T-cell findings are also relevant. In CLL, previous studies have demonstrated quantitative and functional abnormalities in T cells, including chronic activation, exhaustion, impaired immune synapse formation, and expansion of dysfunctional cytotoxic populations [6,8-13]. Inversion of the CD4/CD8 ratio has also been associated with disease biology and prognosis in some studies [10-13]. In this context, the observed differences in CD4-positive T-cell count, CD8-positive T-cell count, and the CD4/CD8 ratio across B-CLPD subtypes suggest that routine lymphocyte subset data may provide supportive information regarding immune disturbances in these disorders [6,8-13].

The relatively higher T-cell and NK-cell proportions observed in HCL were also notable. Classical HCL often presents with cytopenias and splenomegaly rather than marked circulating B-cell predominance, which may partly explain the relatively greater contribution of background lymphocyte subsets in peripheral blood [2,4]. In addition, HCL is strongly dependent on the bone marrow and splenic microenvironment for tumor-cell survival and retention [17-19]. Therefore, the pattern observed in this study may reflect both a lower circulating tumor burden and the contribution of tissue-based microenvironmental support in HCL [17-19].

## Conclusions

Lymphocyte subset profiles differed across B-CLPD subtypes, with the most apparent variation observed in peripheral blood. Differences involved the B-cell, T-cell, and NK-cell compartments, as well as CD4-positive and CD8-positive T-cell measures and the CD4/CD8 ratio. Bone marrow comparisons did not demonstrate statistically significant subtype-related differences, and interpretation was limited by small subgroup sizes. Routine lymphocyte subset data may therefore provide supportive contextual information during flow cytometric assessment but should not replace established lineage-specific and subtype-specific diagnostic markers.
